# When a Circle Becomes the Letter O: Young Children’s Conceptualization of Learning and Its Relation With Theory of Mind Development

**DOI:** 10.3389/fpsyg.2020.596419

**Published:** 2021-01-14

**Authors:** Zhenlin Wang, Douglas A. Frye

**Affiliations:** ^1^Department of Psychology, The Education University of Hong Kong, Tai Po, Hong Kong; ^2^Graduate School of Education, University of Pennsylvania, Philadelphia, PA, United States

**Keywords:** theory of mind, learning concept, knowledge state change, learning intention, epistemic egocentrism

## Abstract

In two independent yet complementary studies, the current research explored the developmental changes of young children’s conceptualization of learning, focusing the role of knowledge change and learning intention, and its association with their developing theory of mind (ToM) ability. In study 1, 75 children between 48 and 86 months of age (*M* = 65.45, *SD* = 11.45, 36 girls) judged whether a character with or without a genuine knowledge change had learned. The results showed that younger children randomly attributed learning between genuine knowledge change and accidental coincidence that did not involve knowledge change. Children’s learning judgments in familiar contexts improved with age and correlated with their ToM understanding. However, the correlation was no longer significant once age was held constant. Another sample of 72 children aged between 40 and 90 months (*M* = 66.87, *SD* = 11.83, 31 girls) participated in study 2, where children were asked to judge whether the story protagonists intended to learn and whether they eventually learned. The results suggested that children over-attributed learning intention to discovery and implicit learning. Stories with conflict between the learning intention and outcome appeared to be most challenging for children. Children’s intention judgment was correlated with their ToM understanding, and ToM marginally predicted intention judgment when the effect of age was accounted for. The implication of the findings for school readiness was discussed. Training studies and longitudinal designs in the future are warranted to better understand the relation between ToM development and children’s learning understanding.

## Introduction

From an early age, discovery learning is important for children’s cognitive development. Gopnik and Wellman’s (2012) probabilistic learning model proposes that infants and young children’s discovery of causal structures based on statistical information gained from exploration and observation is the driving force for cognitive development. However, little is known about children’s understanding of the concept of learning. Do they understand that learning something means acquiring new knowledge? Do they understand that some forms of learning are intentional while others are not? The conceptualization of learning is critical for children’s epistemological thinking (Kuhn, 2000) and affects the outcome of actual learning (Jeong and Frye, 2018a). Focusing on children’s understanding of how the mind works, theory of mind (ToM) research breaks ground for its inquiry into the origins of understanding the mental characteristics of teaching and learning (Kruger and Tomasello, 1996; Olson and Bruner, 1996). In two independent yet complementary studies, the current research explores developmental changes in young children’s conceptualization of learning, focusing on knowledge change and learning intention, and their associations with the development of ToM.

## A Theory of Mind Framework for the Conceptualization of Learning

Although there is evidence that young children, even infants, have an implicit awareness of others’ mental states and can use that information to facilitate their own learning (e.g., Sabbagh and Baldwin, 2001; Birch and Bloom, 2002; Saylor and Troseth, 2006; Harris, 2012), little is known about children’s explicit understanding of the mental activities and processes in learning. Language as Vygotsky’s *psychological tools* (as cited in Fini and Borghi, 2019) functions as an important mechanism in the acquisition of the abstract concepts such as learning. Fini and Borghi (2019) argue that abstract concepts evoke the metacognitive feeling that our knowledge is not sufficient, and we need to learn from more informative others. Infants utilize psychological tools such as imitation, turn-taking, and shared attention to communicate with others and seek help. With the emergence of language, however, abstract language could function as a social tool in metacognition, likely through inner speech (Borghi et al., 2018). Inner speech helps children to retrieve exemplar information, reflect on the meaning of the word, reconstruct the linguistic explanation, and predict what is needed to learn from other sources.

The mental awareness of agency, representational ability, and time perspective are essential to the understanding of learning. Realizing “self as an active cognitive agent and as the causal center of one’s own cognitive activity” (Flavell, 1987, p. 26) might be one of the early ToM achievements that contribute to children’s acquisition of the concept of learning. The distinction between self and others enables children to appreciate others as cognitive agents too. Representational ability gives rise to the awareness of mentality and its fluidity (Perner, 1991). The development of episodic memory (Naito, 2003) and the ability to mentally travel from one time point to another (Atance and Meltzoff, 2005; Atance and O’Neill, 2005; Busby and Suddendorf, 2005) further enable children to appreciate the knowledge state change in the learning process.

While ToM research has primarily focused on children’s understanding of false belief, recent advances include children’s understanding of other epistemic processes such as knowing, remembering, and understanding (Louca, 2019). These topics overlap with those scrutinized in the area of metacognition. Although they share similar research questions, metacognition and ToM research differ in the multiple ways. Research on metacognition focuses on how metacognitive knowledge and regulation affect cognitive achievement in school-aged children. ToM research, on the other hand, mainly focuses on the conceptual underpinnings of these abilities during preschool years. Furthermore, metacognition directs one’s own learning, whereas ToM helps children to understand other people’s mental states (Lockl and Schneider, 2007; Proust, 2012).

Focusing on early development in young children, recent studies have demonstrated that children’s developing ToM is associated with their understanding of knowledge state and intention in the context of teaching (Ziv and Frye, 2004; Frye and Ziv, 2005; Jeong and Frye, 2018b). Children are sensitive to the teacher’s knowledge state in their learning. They choose more knowledgeable informants to learn from (e.g., Sabbagh and Baldwin, 2001; Birch and Bloom, 2002; Harris, 2012). They also demonstrate better learning performance when the informant is knowledgeable compared to ignorant (Jeong and Frye, 2018b). ToM development also contributes to children’s own teaching. Baer and Friedman (2018) asked 4–6-year-old children to describe objects to a listener who was either knowledgeable about the topic or ignorant. Children of all ages were less likely to mention specific facts to a listener who was ignorant of the topic compared with one who was knowledgeable. Older children were also more likely to mention general facts to a knowledgeable listener compared to an ignorant one. Bass et al. (2019) found that children who passed the false belief understanding tasks were more likely to select pedagogical evidence to correct other’s false belief in their teaching. They also found that training children’s pedagogical evidence selection improved their ToM, indicating a reciprocal relationship between ToM and teaching and learning experiences. Jeong and Frye (2018a) found that when children were explicitly told the intention of a teaching event in the direct instruction condition, their intention understanding significantly contributed to the learning outcome. However, this effect was not present in the indirect condition where children were simply told that they were going to play a game without explicit labeling of the teaching intention.

Learning has served as a central construct in psychology, education, neuroscience, artificial intelligence, among many other disciplines (Barron et al., 2015). A full definition of learning is beyond the scope of the current paper; however, a working definition specifies that learning requires enduring changes in knowledge that result from experience (Barron et al., 2015). There are two implications of this definition; first, learning must involve a change in knowledge, and second, the change can either be intentional or unintentional. Knowledge here refers to both declarative knowledge and procedure knowledge, or skills. Knowledge state change involves both updating or gaining descriptive knowledge (i.e., knowing *that*) and gaining new skills (i.e., knowing *how*). Jeong and Frye (2020) recently investigated young children’s understanding of learning as a knowledge-based concept. Children were asked to judge whether they themselves or someone else had learned something new after comparing the knowledge state difference before and after the learning event. The study found that a concept of learning based on knowledge change developed during early childhood: 3-year-olds did not think learning involved a change in knowledge or skill, but 5-year-olds did.

Despite the basic understanding that learning requires a change in knowledge state, there are still unanswered questions about other aspects of children’s concept of learning. For example, when and how do children understand that learning involves an enduring mental representational change? Do children’s own knowledge states affect their judgments of others’ learning? Do children understand that learning can take place with or without intention? Finally, is children’s ToM development related to their understanding of learning?

## Learning as Mental Representational Change

Psychological explanations are essential for children’s teaching and learning (Wellman and Lagattuta, 2004). Young children’s spontaneous utterances about *learning* and *teaching* increase between the ages of 3 and 5 years (Bartsch et al., 2003). However, their narratives about learning and teaching tend to focus on the behavioral terms instead of mental state terms. For example, young children describe learning as “listen to the teacher;” “sit up… so you can learn more,” instead of “thinking” (Thorpe et al., 2004). Young children describe teaching as “showing,” while older children who have acquired false belief understanding describe teaching as “telling” (Astington and Pelletier, 1996). Pramling (1988) characterized young children’s initial concept of learning as that of behavioral change, i.e., *learning to do*. At this point, the content to be learned is usually a skill, an activity, or a behavior. With age, children proceed to a higher level understanding of learning as representational change, i.e., *learning to know*. They begin to talk about facts or knowledge as intellectual properties. Only in elementary school do children begin to appreciate that learning changes thinking itself, i.e., *learning to understand*.

Sobel and Letourneau (2018) presented 3–5-year-olds with stories of a character who either learned something through own exploration or from explicit instructions given by others. Younger children could correctly report learning from exploration, but they underestimated learning from direct instruction. In fact, they tended to attribute learning in both types of stories to actions. Older children were more likely to differentiate the two types of learning and correctly identify the knowledge source. The authors concluded that younger preschoolers’ action-oriented learning concept showed that they were yet to develop a metacognitive understanding of how learning occurred.

The first goal of the current study was to explore when children understand that learning requires genuine knowledge change. In other words, when do they appreciate that learning is more than just a change in behavior, but also a mental representational change? To answer this question, children were given a new task which featured a person who did not know how to write a letter O, but nevertheless learned how to draw a small circle perfectly. Children were asked whether the person had learned to write the letter O or not. To understand that the behavior of drawing a circle is not enough for learning to happen requires a mental representational concept of learning. The behavioral change without mental representational change is not replicable or enduring. Learning only occurs when the new representational meaning of the circle is acquired.

## Egocentrism

Young children tend to erroneously assign their own knowledge and belief to others. In the unexpected content false belief task (Gopnik and Astington, 1988), after seeing the real content of a misleading container firsthand, 3-year-old children could not understand that a naïve protagonist who had not seen the content would not know what was really in it. Similarly, children in the unexpected location false belief task (Wimmer and Perner, 1983) witnessed an object being moved from one location to another while the story protagonist was away. However, young children consistently claimed that the protagonist would look for the object in the new location, even though they knew that he had not seen the location change.

Arising from self-agency, this self-centered perspective has been extensively researched under various labels, such as egocentric perspective taking (Piaget and Inhelder, 1956), curse of knowledge (Camerer et al., 1989; Birch and Bloom, 2003; Birch, 2005), and epistemic egocentrism (Royzman et al., 2003). For example, Birch and Bloom (2003) tested 3–5-year-old children who either knew or did not know what was inside a toy. They were then asked to judge whether a character knew the content. The results revealed that when 3- and 4-year-old children were ignorant of the content, they were more accurate at judging other’s knowledge state. In contrast, when young children knew what was inside the toy, they overestimated other’s knowledge, as if their judgments were “cursed” by their own knowledge. The magnitude of the bias decreased with age, indicating younger children were more prone to the curse of knowledge. Interestingly enough, when the other party was familiar with the toy, there were no differences in children’s judgment of whether the other party knew the content or not between the child-knowledgeable and the child-ignorant conditions across the age groups. Even the youngest children were able to judge the informed other party knew what was inside of the toy, suggesting children were indeed able to take other people’s perspectives; they were only biased by their own knowledge when making judgment about someone who was more ignorant than themselves.

Given the epistemic egocentrism, would children’s own previous knowledge affect their judgment of others’ learning? The second goal of the study was to explore the effect of egocentrism on children’s judgments of learning. If children are familiar with the material being learned, would they be more prone to say that others have learned it too? On the other hand, would children’s learning judgments be more accurate if the learning content is entirely novel to them and they do not have any previous knowledge to interfere with their judgment? Finally, if children show an egocentric bias, does it affect children of different developmental stages equally? To our knowledge, this is the first study examining the potential impact of epistemic egocentrism on children’s concept of learning.

## Intention to Learn

Unlike teaching, which is “an intentional activity to increase the knowledge (or understanding) of another” (Ziv and Frye, 2004, p. 458), learning does not have to be intentional. While intentional or deliberate learning is often associated with optimal learning outcomes, learning could happen without intention, such as in discovery learning and implicit learning. It has been found that young children generally have difficulty understanding when a desired outcome is achieved by coincidence (Phillips et al., 1998). For example, preschoolers fail to recognize that certain bodily functions, such as knee-jerk reactions or sneezes, are unintentional (Lang and Perner, 2002; Montgomery and Lightner, 2004). Young children also find it difficult to judge whether an act is moral or not based on intentions. Studies have documented that around 4–5 years of age, children’s moral judgment goes through an outcome based to intent based shift (Cushman et al., 2013; Margoni and Surian, 2016, 2017, 2020; Nobes et al., 2017).

In studies on children’s understanding of teaching intentions (Frye and Ziv, 2005; Ziv et al., 2008), 3- and 5-year-olds were told stories about an instance of imitation where the teacher was not aware of the presence of the learner. Three-year-olds reported that the teacher tried to teach even without knowing the learner was there. Only 5-year-olds who passed the false belief task could distinguish the intention to teach from the intention to learn in the imitation task. Another story described an instance of a hidden teaching intention, in which a teacher did not make the teaching intention explicit; instead, she specified that she was going to “play a game” with the children. Three-year-olds failed to detect the teaching intention embedded in an educational game; only 5-year-olds could tell that the teacher was really trying to teach. It seems at least in the case of teaching, young children found it difficult to understand an intention that was not explicitly stated, or in conflict with the teaching and learning outcome.

Sobel et al.’s (2007, study 2) has examined children’s understanding of motivational mental states in learning, including desire, attention, and intention. In this study, 4- and 6-year-olds were told stories of children learning a song from a teacher. Each story presented two mental states that were either consistent or inconsistent with each other. For example, a character who had the desire to learn might be either paying attention to the teacher’s demonstration (Desire+/Attention+) or not (Desire+/Attention−). Children were asked whether the character learned the song and why. Children performed well in the consistent stories, but not in the inconsistent ones. Four-year-olds were more likely than 6-year-olds to judge that the character who wanted to learn but did not pay attention nonetheless learned. Young children’s performances on the inconsistent stories were not different from chance level. The authors argued that 4-year-olds tended to judge whether someone learned based on desire, whereas 6-year-olds were more likely to integrate desire, intention, and attention in learning together.

By posing the task questions in an open-ended manner (“Did the person learn how to sing the song?”), the design of this study assumed a causal relation between the motivational mental states and the learning outcomes, which is not always the case. As discussed, learning does not have to be intentional; and even intentional learning does not always bring out the intended outcome. In other words, the design of the study implicitly defined learning as a direct outcome of motivation, instead of representational knowledge change. The consequence of such is especially problematic in the inconsistent stories. The answer to the question of whether the character learned the song in those stories is rather arbitrary. It is equally possible for one to learn a song or fail to do so in the inconsistent stories, which could explain children’s chance level performance.

The third goal of the current study was to explore children’s understanding of intention to learn and its correlation with their developing ToM. Different from previous studies, purposely designed tasks in the current study presented scenarios with various learning intentions coupled with either successful or failed learning outcomes, such as discovery learning when someone learned to make the color green by accidentally mixing blue paint and yellow paint; or implicit learning when someone learned a song simply by overhearing it, in order to explore whether and when children understand that having an intention to learn is not necessary for learning to occur.

## The Present Study

The two studies reported in the current paper were part of the doctoral dissertation of the first author (Wang, 2010). Study 1 investigates when children understand that learning is a mental representational change instead of a behavioral one. It also examines whether children’s own knowledge state affects their judgments of others’ learning. Study 2 explores children’s understanding of learning intention in different learning scenarios. In addition, both studies scrutinize the association between children’s comprehension of the learning concept and their ToM development.

## Study 1: Knowledge Change and Epistemic Egocentrism

### Method

#### Participants

Jeong (2018) reported correlations between ToM and judgment of whether learning occurred ranging from 0.287 to 0.342. *A priori* power analysis was conducted in G^∗^Power (Faul et al., 2009) adopting a conservative 0.287 as the correlation between ToM and learning judgment. Due to the one-directional nature of the correlation, one-tailed test was used with the alpha level set at 0.05. The results showed that 73 participants were required to achieve 80% power. Seventy-five children (36 girls) aged between 48 and 86 months from two preschools and two primary schools representing a wide range of social economic status neighborhoods in Hong Kong were recruited, including 25 4-year-olds (*M* = 52.80, *SD* = 3.22, 11 girls), 25 5-year-olds (*M* = 64.52, *SD* = 3.81, 12 girls), and 25 6-year-olds (*M* = 79.04, *SD* = 4.38, 13 girls). All children were fluent in Cantonese.

#### Measures and Procedure

The study protocol was reviewed and approved by a local university’s Human Research Ethics Committee. Parents signed informed consents and children gave oral consents for participating in the study. Children individually participated in six learning tasks purposely designed for this study and three ToM tasks in one or two sessions of 15 min each in a quiet room in school with a trained experimenter. The tests were administered in Cantonese. The sequence of the learning tasks and the ToM tasks was counterbalanced.

##### The learning task

The purposely designed learning task in this study included three familiar content stories and three unfamiliar content stories. In each of these six stories, there were two characters who both produced a symbol, such as drawing a circle. One of the characters was told by the teacher the representational meaning of the symbol, hence acquiring a genuine knowledge change in the process, while the other failed to realize the representational meaning of the symbol and therefore did not achieve genuine knowledge change. The learning contents of the six stories were designed to include both knowledge that children were familiar with and novel knowledge that children would not possess, such as a symbol from a foreign language. At the end of each story, children were asked two memory control questions to check their comprehension of the stories, and a learning question to judge which character *learned* the knowledge. Stories used in the Study 1 are available in Supplementary Material.

The six stories were presented to children in a random order with props including paper, pencil, wooden letter blocks, and toy figurines. Children were given one point for each correctly answered learning question. The maximum scores for the familiar and unfamiliar learning tasks were both 3.

##### ToM measure

Theory of mind was measured using the *Knowledge Access* task, the *Contents False Belief* task, and the *Explicit False Belief* task as described in Wellman and Liu’s (2004) ToM scale. The three tasks were selected in the current study because they measure the epistemic mental states of knowledge, belief, and false belief that are closely related to learning. The sequence of the three tasks was randomized in administration. Children scored 1 point for passing each task, making the maximum score for the ToM tasks 3 points.

### Results

Five 6-year-old children did not finish the learning tasks. Listwise deletion was adopted in the following analysis. There were no significant differences between boys’ and girls’ performances, *t*(68) = 0.084, and *p* = 0.933 for unfamiliar tasks, with *M* = 1.94, *SD* = 1.03 for girls and *M* = 1.92, *SD* = 1.01 for boys; and *t*(68) = 0.403, *p* = 0.688 for familiar tasks, with *M* = 1.91, *SD* = 0.95 for girls and *M* = 1.81, *SD* = 1.08 for boys.

**FIGURE 1:**
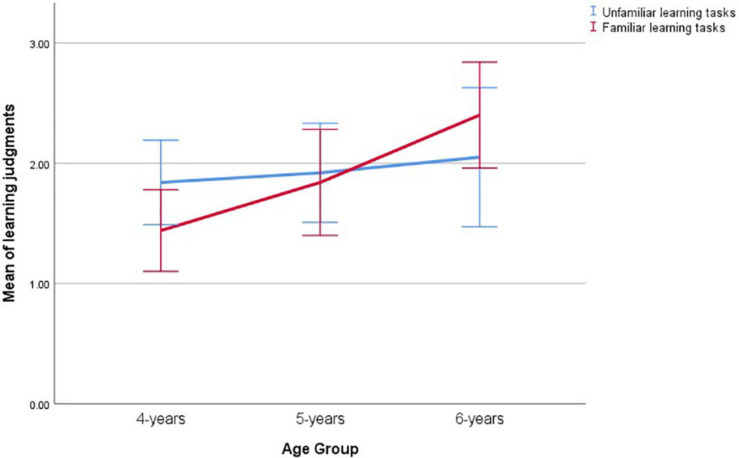
Children’s learning judgments in study 1 across age groups (error bars representing 95% CIs).

Table 1 presents the descriptive statistics for the variables in study 1. Figure 1 shows the developmental changes in children’s learning judgment in familiar and unfamiliar tasks by age group, with the error bars representing 95% CIs. Repeated measures multivariate analysis with familiarity as a within-subject factor and age as between-subject factor showed that familiarity did not significantly affect children’s learning judgment, *F*(1,67) = 0.135, *p* = 0.714, η^2^ = 0.002, Cohen’s *f* = 0.04. There were no significant differences among age groups either, *F*(2,67) = 2.597, *p* = 0.082, η^2^ = 0.072. However, the interaction between familiarity and age group did occur, *F*(2,67) = 3.126, *p* = 0.045, η^2^ = 0.089, Cohen’s *f* = 0.31. Four-year-old children performed marginally better in unfamiliar tasks (*M* = 1.84, *SD* = 0.85) than in familiar tasks (*M* = 1.44, *SD* = 0.82), *t*(24) = 1.732, *p* = 0.096, Cohen’ *d* = 0.48 (Cohen, 1988). Six-year-old children, however, did slightly better in the familiar tasks (*M* = 2.40, *SD* = 0.94) than the unfamiliar tasks (*M* = 2.05, *SD* = 1.23), Cohen’ *d* = 0.32, although the difference was not statistically significant, *t*(19) = −1.584, *p* = 0.130.

**TABLE 1:** Descriptive statistics of Study 1.

	ToM	Familiar learning tasks	Unfamiliar learning tasks
	Mean ± SD [95% CI]	Mean ± SD [95% CI]	Mean ± SD [95% CI]
4-year-old	0.92 ± 0.76 [0.61, 1.23]	1.44 ± 0.82 [1.10, 1.78]	1.84 ± 0.85 [1.49, 2.19]
5-year-old	1.44 ± 0.92 [1.06, 1.82]	1.84 ± 1.07 [1.40, 2.28]	1.92 ± 1.00 [1.51, 2.33]
6-year-old	2.16 ± 0.90 [1.79, 2.53]	2.40 ± 0.94 [1.96, 2.84]	2.05 ± 1.23 [1.47, 2.63]
Total	1.51 ± 0.99 [1.28, 1.73]	1.86 ± 1.01 [1.62, 2.10]	1.93 ± 1.01 [1.69, 2.17]

**N* = 70.*

ANOVA with age as between-subject factor predicting learning judgment in familiar tasks showed that the means of the learning judgments in the familiar tasks increased with age, *F*(2,67) = 5.693, *p* = 0.005, η^2^ = 0.145, Cohen’s *f* = 0.41. Four-year-olds performed at chance level when they were asked which character learned in the story, *t*(24) = −0.366, *p* = 0.718. Scheffé *post hoc* comparisons further demonstrated that 6-year-olds (*M* = 2.16, *SD* = 0.90) outperformed 4-year-olds (*M* = 1.44, *SD* = 0.82) in their learning judgment in the familiar tasks, *p* = 0.005, Cohen’ *d* = 0.84. On the contrary, the means of the learning judgments in the unfamiliar tasks were unchanged with age, *F*(2,67) = 0.235, *p* = 0.791; η^2^ = 0.007, Cohen’s *f* = 0.08, although the overall mean was above chance level, *t*(69) = 3.544, *p* = 0.001.

Children’s performance on the ToM tasks increased with, *F*(2,72) = 13.079, *p* < 0.001, η^2^ = 0.266, Cohen’s *f* = 0.60. While children’s ToM performance was not correlated with their learning judgments in the unfamiliar tasks, *r* = 0.034, *p* = 0.782, the correlated between ToM performance and learning judgments in the familiar learning tasks was significant, *r* = 0.288, *p* = 0.016; but the correlation became statistically non-significant once age was taken into consideration, *r* = 0.121, *p* = 0.322. Liner hierarchical regression was performed to predict learning judgment in familiar tasks with age entered in the first step and ToM entered in the second step. Only age significantly predicted children’s learning judgment, explaining 17.4% of the variance, *F*(1,68) = 14.365, *p* < 0.001. ToM explained extra 1.3% of the variance, *F*(1,67) = 1.049, *p* = 0.310. Regression coefficients are presented in Table 2.

**TABLE 2:** Hierarchical linear regression predicting learning judgment in familiar tasks in study 1.

Model		Unstandardized coefficients		Standardized coefficients	*t*	*P*	95% Confidence interval for B	
		B	SE	Beta			Lower bound	Upper bound
1	(Constant)	−0.623	0.664		−0.939	0.351	−1.948	0.701
	Age	0.039	0.010	0.418	3.790	0.000	0.018	0.059
2	(Constant)	−0.478	0.678		−0.705	0.483	−1.832	0.876
	Age	0.033	0.011	0.361	2.929	0.005	0.011	0.056
	ToM	0.131	0.128	0.126	1.024	0.310	−0.124	0.387

**N* = 70.*

### Discussion

Study 1 found that children’s learning judgment in familiar tasks improved significantly with age between 4 and 6 years. Four-year-old children attributed learning randomly between somebody who gained knowledge and somebody who performed an accidental coincidence that did not involve mental state change when they were familiar with the learning contents. Six-year-old children could correctly judge that accidental coincidence without representational change did not count as learning, and attribute learning only to situations where genuine knowledge change happened. Contrary to that in the familiar tasks, children’s learning judgment did not improve with age in the unfamiliar tasks. Four-year-old children performed better in the unfamiliar tasks comparing to familiar tasks, while 6-year-old children did better in the familiar tasks. ToM was associated with children’s learning judgment, but only when the learning contents were familiar. Furthermore, this association was largely driven by age. Once age was accounted for, the correlation between ToM and learning judgment in the familiar learning tasks was no longer significant.

The finding of younger children’s indiscriminative learning attribution adds to the earlier reports on children’s immature learning concept. It is worth noting that although it was already explicitly stated at the end of the story that one character now *knew* that was how to write a letter O while the other character still *did not know*, children were not making their learning judgment by simply relying on this statement. If they were indeed echoing this statement, they should have only attributed learning to the character who *knew*. The fact that younger children randomly attributed learning regardless whether or not the character knew its representational meaning indicates that they were not relying on knowledge change in their learning judgment.

Echoing Birch and Bloom’s (2003) findings on curse of knowledge, the current result showed that younger children were affected by the familiarity of the learning contents. For 4-year-old children, being familiar with the learning contents themselves hindered their learning judgments. In contrast, being familiar with the content actually helped 6-year-old children to realize the protagonist’s knowledge state had changed from being ignorant to being knowledgeable like themselves, although the effect was not statistically significant. The transition from *everybody should know what I know* to *you now learned what I know* reflects a developing self-other distinction that bridges mental state understanding of self (metacognition) and that of others (ToM). Although children’s performance on the unfamiliar learning tasks was above chance level across age groups, even 6-year-olds’ answers were still not perfect, indicating that by the time of school entry children were yet to develop a mature understanding of the concept of learning when facing novel tasks. Reflecting on their own knowledge state might be helpful for older children to develop an appreciation of how learning occurs in others.

The current results demonstrated a preliminary correlation between children’s learning judgment and their developing ToM, consolidating Jeong and Frye’s (2018b) finding. However, ToM was not a significant predictor to children’s learning judgment in familiar tasks when the effect of age was accounted for, indicating that the changes in learning judgment were mostly driven by maturation.

Knowledge change is a necessary and sufficient condition for learning. Intention, on the other hand, is neither, even though it plays an important role in learning. Learning takes place as long as there is genuine knowledge change, no matter whether it is done on purpose or occurs as an accidental discovery. Study 2 explores how well young children understand the complex mechanism of intention’s involvement in learning, especially when there is a conflict between the learning intention and its outcome.

## Study 2: Learning Intention in Discovery and Implicit Learning

### Method

#### Participants

There are no known studies reporting the correlation between ToM and learning intention judgment. Jeong and Frye (2018a) reported a correlation of 0.374 between ToM and teaching intention judgment, which was adopted here as reference in power analysis. *A priori* power analysis was conducted in G^∗^Power (Faul et al., 2009). Due to the one-directional nature of the correlation, one-tailed test was used with the alpha level set at 0.05. The result showed that 42 participants were required to achieve 80% power. Severn-two children (31 girls) aged between 40 and 90 months were recruited from a preschool and a primary school representing a wide range of social economic status neighborhoods in Chong Qing, China. There were 24 children aged 4 years and younger (*M* = 54.08, *SD* = 3.99, 10 girls), 24 5-year-olds (*M* = 65.50, *SD* = 2.99, 11 girls), and 24 6 years and older (*M* = 81.04, *SD* = 5.75, 10 girls). All children were fluent in Mandarin Chinese.

#### Measures and Procedure

The study protocol was reviewed and approved by an overseas university’s Institutional Review Board. Parents signed informed consents and children gave oral consents for participating in the study. Individual children participated in four learning tasks purposely designed for this study and three ToM tasks within 30 min in a quiet room in school with a trained experimenter. The tests were administered in Mandarin Chinese. The sequence of the learning tasks and the ToM tasks was counterbalanced. Both the four learning tasks and the three ToM tasks were administered in random order.

##### The learning tasks

Four learning intention stories were purposely developed for this study in a two-by-two design. They involved two levels of learning outcomes: positive and negative; and two levels of learning intentions: learning without intention and learning with a resistance intention. Table 3 outlines the task specifications of study 2. There were no conflicts between the learning intentions and learning outcomes in the *failed learning* or *resistance to learning* tasks. The protagonists in these two stories either did not intend to learn, or resisted learning, and ended up not learning. In contrast, conflicts were presented in the other two stories. The protagonist in the *discovery learning* story discovered how to mix color green from other colors by accident. The one in the *implicit learning* story learned a song which he actually tried very hard not to learn after overhearing it. Stories used in study 2 are available in Supplementary Material.

**TABLE 3:** Task specifications of study 2.

	Learning intention	Learning outcome
Discovery learning	Negative	Yes
Failed learning	Negative	No
Implicit learning	Resistance	Yes
Resistance to learning	Resistance	No

The stories were presented to children with figurines, drawings, color paints, and brushes for demonstration. The experimenter read each story to children first, and then asked two control questions about the characters’ knowledge state before and after the learning event. In case children answered any of the control questions incorrectly, their responses on that story were excluded from the analyses. Two task questions on the learning intention and learning outcome followed. Children scored 1 point for each correctly answered task question, making the maximum scores for both the intention judgment and the learning judgment 4 points.

##### ToM measures

The ToM tasks were identical to the ones in study 1.

### Results

Seven children answered at least one of the knowledge control questions incorrectly. Their responses on that story were excluded from the analyses. Occasionally children answered “don’t know” to the intention question or the learning question, which was treated as incorrect answer. Independent-samples Mann–Whitney *U*-tests showed that intention and learning judgment distributions in boys and girls did not differ significantly, standardized Mann–Whitney *U* ranging from −1.070 to 0.614, *p* ranging from 0.285 to 1.000.

**FIGURE 2:**
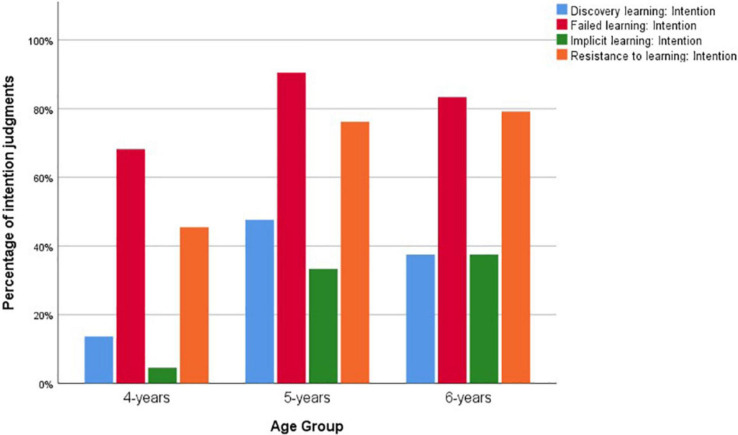
Children’s intention judgments in study 2 across age groups.

Table 4 shows the descriptive statistics of study 2. Figure 2 shows children’s intention judgment across age groups in the individual learning tasks. Cochran’s *Q* test was adopted to compare within-subject binary intention judgments. The result indicated that there were differences in children’s responses across the four learning tasks on the intention question, *x^2^* = 59.948, *df* = 3, *p* < 0.001. Children performed significantly better in tasks without conflict between the learning intention and learning outcome, i.e., the *failed learning* and the *resistance to learning* stories, than those with a conflict, i.e., the *discovery learning* and the *implicit learning* stories. There were significant differences between *discovery learning* and *failed learning* intentions, McNemar’s *x^2^* = 29.257, *df* = 1, *p* < 0.001, and between *implicit learning* and *resistance to learning* intentions, McNemar’s *x^2^* = 18.27, *df* = 1, *p* < 0.001. However, even 5- and 6-year-old children’s intention judgments in the *discovery learning* and the *implicit learning* stories were not significantly different from chance level, *t*(45) = −1.185, *p* = 0.242 for the *discovery learning* story, and *t*(45) = −2.002, *p* = 0.052 for the *implicit learning* story, respectively.

**TABLE 4:** Descriptive statistics of study 2.

	ToM	Sum of intention judgments	Sum of learning judgments
	Mean ± SD [95% CI]	Mean ± SD [95% CI]	Mean ± SD [95% CI]
4-year-old	0.92 ± 0.92 [0.52, 1.31]	1.32 ± 0.78 [0.97, 1.66]	3.46 ± 0.83 [3.11, 3.81]
5-year-old	1.96 ± 0.71 [1.65, 2.26]	2.48 ± 0.98 [2.03, 2.92]	3.55 ± 0.74 [3.22, 3.87]
6-year-old	2.05 ± 0.76 [1.69, 2.41]	2.38 ± 1.01 [1.95, 2.80]	3.63 ± 0.71 [3.32, 3.93]
Total	1.61 ± 0.95 [1.28, 1.84]	2.06 ± 1.06 [1.80, 2.32]	3.54 ± 0.76 [3.36, 3.72]

**N* = 70.*

ANOVA predicting intention judgment with age as between-subject factor showed that there was a significant age effect on the sum of the four intention judgments, *F*(2,64) = 10.425, *p* < 0.001, η^2^ = 0.246, Cohen’s *f* = 0.57. Independent-samples Kruskal–Wallis tests showed that there were significant age differences in two of the four intention judgments, with *x^2^* = 6.028, *df* = 2, *p* = 0.049 for the *implicit learning* story, and *x^2^* = 9.258, *df* = 2, *p* = 0.010 for the *resistance to learning* story.

**FIGURE 3:**
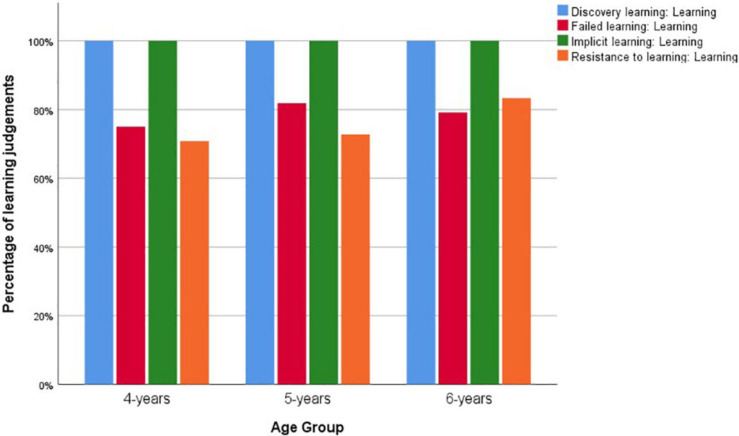
Children’s learning judgments in study 2 across age groups.

Figure 3 shows children’s learning judgments across age groups in the individual tasks. All children answered the learning questions correctly in tasks with positive learning outcomes, i.e., the *discovery learning* and the *implicit learning* stories. Although the protagonists’ knowledge states were stated explicitly at the end of the stories, children’s responses in the tasks with negative learning outcomes, i.e., the *failed learning* and the *resistance to learning* stories, were less than perfect. Children’s performances on the four learning questions differed significantly, Cochran’s *Q* test showed *x*^2^ = 41.838, *df* = 3, *p* < 0.001.

ANOVA with age as between-subject factor revealed that there were significant age related differences in the ToM score, *F*(2,64) = 13.931, *p* < 0.001, η^2^ = 0.303, Cohen’s *f* = 0.67, with the 6-year-old children performing the best. Children’s ToM was not correlated with their learning judgments, *r* = 0.032, *p* = 0.807. The sum of the four intention judgments was significantly correlated with the ToM score, *r* = 0.358, *p* = 0.004; however, the correlation was no longer significant when age was held constant, *r* = 0.155, *p* = 0.232. Liner hierarchical regression was performed to predict intention judgment with age entered in the first step and ToM entered in the second step. Age significantly predicted children’s intention judgment in model 1, explaining 12.0% of the variance, *F*(1,55) = 7.493, *p* = 0.008. When ToM was entered in the regression, however, age was no longer a significant predictor. ToM was a marginally significant predictor for children’s intention judgment, with a small to moderate unstandardized coefficient of 0.246, explaining extra 4.4% of the variance, *F*(1,54) = 2.856, *p* = 0.097. Regression coefficients are presented in Table 5.

**TABLE 5:** Hierarchical linear regression predicting intention judgment in study 2.

Model		Unstandardized coefficients		Standardized coefficients	*t*	*p*	95% Confidence interval for B	
		B	SE	Beta			Lower bound	Upper bound
1	(Constant)	0.084	0.699		0.120	0.905	−1.316	1.485
	Age	0.029	0.010	0.346	2.737	0.008	0.008	0.050
2	(Constant)	0.347	0.705		0.492	0.625	−1.066	1.760
	Age	0.019	0.012	0.226	1.579	0.120	−0.005	0.043
	ToM	0.246	0.146	0.242	1.690	0.097	−0.046	0.539

**N* = 57.*

### Discussion

Study 2 found that children’s understanding of learning intention improved with age. However, they did over-attribute learning intention in the discovery learning and implicit learning tasks, in which the intentions were in conflict with the learning outcomes. Even at 5 and 6 years of age, children’s performances on the intention judgments in these two tasks were still at chance level. In other words, when discovery and implicit learning were successful, children did not recognize the learning was unintentional. Children’s judgment of learning intention was correlated with their developing ToM with a moderate effect size. ToM marginally predicted children’s intention judgment when the effect of age was accounted for.

Human actions are assumed intentional until proven otherwise (Rosset, 2008). The focus of intention understanding is not how to infer intention, but how to inhibit it. Children over-attributed learning intention to discovery and implicit learning in the current study. They seemed to assume that if somebody eventually learned something, he or she must have intended to do so. This finding was consistent with previous reports on children’s intention over-attribution in case of voluntary bodily function and pretense (e.g., Lillard, 1993; Lang and Perner, 2002; Montgomery and Lightner, 2004). It was also in line with children’s over-attribution of teaching intention to imitation (Frye and Ziv, 2005; Ziv et al., 2008; Jeong and Frye, 2018a).

The result provided preliminary evidence suggesting that ToM development during 4–6 years of age was associated with children’s learning intention attribution. The moderate effect sized zero-order correlation between ToM understanding and children’s learning intention judgments in the current study (*r* = 0.358) was comparable to that between ToM and teaching intention judgment reported by Jeong and Frye (2018a) (*r* = 0.374). Consistent with children’s understanding of the intention to teach (Frye and Ziv, 2005; Ziv et al., 2008), ToM enables children to focus on the motivational mental states leading to learning rather than the behavioral outcome alone. Unlike teaching, which is intentional, intention is neither a necessary nor a sufficient condition for learning to happen. Children with more advanced ToM understanding should be better at detecting the “aha” moment in discovery learning, where the knowledge change comes as a surprise for the learner exactly because of the lack of an initial learning intention. This is the first empirical evidence according to our knowledge on the association between children’s ToM development and their learning intention judgment.

Even though the learning outcomes were explicitly stated in the stories, children found it difficult to entertain the idea that learning could fail. Children’s over-attribution of learning in the failed learning scenarios (*failed learning* and *resistance to learning*) replicated study 1’s finding. Even more so, compared to the characters in study 1 who could perform the action without a mental representational change, no learning actions were mentioned in these scenarios in study 2. A small proportion of children still believed that the characters had learned in these stories. This finding also confirmed Sobel et al.’s (2007, study 2) result that children over-attributed learning with more rigorous research design. It is possible that this phenomenon might indicate a *Yes* bias when children are asked a yes-no question (Fritzley and Lee, 2003; Okanda and Itakura, 2008). Future studies should consider adopting forced choice format in questioning to differentiate these possibilities.

## General Discussion

Theory of mind is a “core human cognition” that is important because it “shapes human thoughts and learning” (Wellman, 2004, p. 2). The current study contributed to our understanding of changes in children’s conceptualization of learning through highlighting the role of knowledge change and intention understanding. At the same time, the study systematically demonstrated the association between children’s learning concept and their developing ToM. Small to moderate zero-order correlations were identified between children’s ToM and their understanding of knowledge change and learning intention.

The current study contributed a new task to identify a shift in children’s understanding that learning involves changes not only in behavior, but more importantly in knowledge state between 4 and 6 years of age. The tasks in study 1 tested children’s understanding that learning to draw a symbol like the letter O requires not just the act of drawing a circle, but also acquiring the representational meaning of the symbol. The responses of the 6-year-olds showed they were beginning to see learning as a change in mental representation. The second study further examined whether 4–6-year-olds appreciate that learning does not necessarily require intention as in the instances of discovery and implicit learning. The findings showed that even the 6-year-olds seemed to over-attribute intention to learning and did not recognize that discovery and implicit learning could occur unintentionally.

The findings suggest that at school entry, 6-year-olds still face challenges in some of the learning comprehension tasks, especially those with a conflict between a learning intention and its outcome. This result is similar to Sobel and Letourneau’s (2015) interview study that found that children’s tendency to define learning as a process improved between 4 and 8 years. It seems that learning concept undergoes a prolonged developmental period beyond early childhood. Sobel and Letourneau further suggested that a process-based learning concept might be related to an interpretative ToM that matures during middle childhood (Carpendale and Chandler, 1996).

Both studies found correlations between learning judgments and ToM understanding. However, the correlational nature of the current study makes it impossible to infer the direction of causation. Wang et al. (2017) examined the correlation between 3- and 6-year-old children’s performance on the ToM tasks and a battery of 16 teaching and learning comprehension tasks in a cross-sectional study with two samples from Hong Kong and the United States. A moderate correlation was found between the two constructs, even after controlling for age and verbal ability in both samples. Comparing competing structural equation models with either construct as predictor, they found that the model with the teaching and learning comprehension as predictor and the ToM as outcome fit the data significantly better than otherwise, indicating that earlier understanding of teaching and learning might be inductive to ToM development.

The relation between ToM development and children’s understanding of teaching and learning might be bidirectional (Davis-Unger and Carlson, 2008; Bass et al., 2019). It is likely that mature mindreading ability facilitates understanding of teaching and learning. It is also possible that exposure to conflicts in perspectives and knowledge differences may enhance children’s understanding that beliefs might be inconsistent with the reality (Wellman and Lagattuta, 2004; Wang et al., 2017). In light of the development of both ToM (e.g., Wang et al., 2016) and epistemological understanding (Burr and Hofer, 2002) beyond early childhood, future longitudinal research should test whether epistemological understanding links earlier mental state awareness to later metacognition knowledge.

The current findings not only enriched ToM research, but more importantly shed light on young children’s metacognitive understanding of the learning. The false belief paradigm in ToM research productively demonstrates children’s over-attribution of knowledge to naive others and themselves. The current research further demonstrates that young children also tend to over-attribute knowledge change and intention in their understanding of learning. These findings open up the possibility that children’s initial understanding of learning may also be an important component of school readiness. According to Astington (1998), mental state understanding helps children to succeed in school through numerous ways, including increasing their representational capacity, language ability, narrative understanding and literacy, intentional learning and objective knowledge, social competence and collaborative learning, as well as in the first steps in scientific reasoning. If part of the success of formal schooling depends on both the teacher and student having some awareness of the overall point of the activity, then the change in children’s understanding should be an advantage for school entry. Future research on the effect of understanding of learning on learning outcomes and school performance is warranted (Jeong and Frye, 2018a, b; Louca, 2019). Training studies focusing on improving children’s mental state understanding for preparation of school entry should be fruitful.

This study has caveats. The learning tasks, especially those with conflict, may have taxed children’s executive functions (Zelazo and Frye, 1997), which were not measured in the current study. Compared to Western children, Asian children develop executive functions earlier, but their ToM development is not equally advanced (Sabbagh et al., 2006; Oh and Lewis, 2008; Wang et al., 2016). By 3.5 or 4 years of age, Asian children have already developed an above-chance level of inhibitory control. However, within a specific culture, inhibitory control still correlates with ToM development (Sabbagh et al., 2006). Future studies should consider measuring children’s executive function to identify the effects of both domain general cognitive ability and specific mental state understanding on children’s conceptualization of learning. Replications of the current findings with diverse samples are needed. Another limitation of the current study is the lack of a linguistic ability test to examine the role of language in children’s learning concept development. ToM development is highly dependent on language ability (Milligan et al., 2007). In light of the important role of abstract concept as psychological tool (Borghi et al., 2018; Fini and Borghi, 2019) in ToM development, future research is warranted to explore how children’s language development facilitates metacognition and the acquisition of abstract concepts like learning.

## Data Availability Statement

The raw data supporting the conclusions of this article will be made available by the authors, without undue reservation.

## Ethics Statement

The studies involving human participants were reviewed and approved by the Human Research Ethics Committee, The Education University of Hong Kong, and the Institutional Review Board, University of Pennsylvania. Written informed consent to participate in this study was provided by the participants’ legal guardian/next of kin.

## Author Contributions

ZW collected and analyzed the data, and drafted the manuscript. DF provided critical revision to the manuscript. Both authors contributed in the study conceptualization and design.

## Conflict of Interest

The authors declare that the research was conducted in the absence of any commercial or financial relationships that could be construed as a potential conflict of interest.
