# Supplementary material for: When a Circle Becomes the Letter O: Young Children’s Conceptualization of Learning and Its Relation With Theory of Mind Development
Source: Front Psychol. 2021 Jan 14;11:596419. doi: 10.3389/fpsyg.2020.596419 (PMC7841392; doi:10.3389/fpsyg.2020.596419)
Supplement: Supplementary file 1 [file Data_Sheet_1.docx]

**When a circle becomes the letter O:**

**Young children’s conceptualization of learning and its relation with theory of mind development**

Zhenlin Wang

Department of Psychology, The Education University of Hong Kong

Douglas Frye

Graduate School of Education, University of Pennsylvania

Author Note: Correspondence concerning this paper should be addressed to: Zhenlin Wang, Department of Psychology, The Education University of Hong Kong, 10 Lo Ping Road, Tai Po, Hong Kong SAR or by email to [zlwang@eduhk.hk](mailto:zlwang@eduhk.hk).

**Supplemental Materials for Online Publication Only**

**Familiar and unfamiliar learning stories in Study 1**

1. **Familiar content: Duel Identity**

Do you know how to write a letter *O*? Can you write a letter *O* for me?

These are Jun and Wei. They cannot write a letter *O*. They just can’t.

Today in school, Jun and Wei each draw a circle: *O*. It looks just like letter *O*, doesn’t it?

Jun shows his circle to the teacher. The teacher says to Jun: “This is a circle, you just drew a nice circle.” Only Jun does not know this is how you write a letter *O*. He does not know this is a letter *O*.

Wei shows her circle to the teacher. The teacher says to Wei: “This is a letter *O*, you just wrote a letter *O*.” Now Wei knows this is how you write a letter *O*. She knows this is a letter *O*.

**Control questions:**

At the end of the story, does Jun know this is how you write a letter *O*?

At the end of the story, does Wei know this is how you write a letter *O*?

**Learning question:**

Who learned how to write a letter *O* today, Wei or Jun?

1. **Familiar content: Copy**

Do you know how to write a letter *Y*? Can you write a letter *Y* for me?

These are Hua and Mei Ran. They cannot write a letter *Y*. They just can’t.

Today in school, Hua and Mei Ran each copy a letter from the board: *Y*. It looks just like letter *Y*, doesn’t it?

Hua shows her letter to the teacher. The teacher says to Hua: “This is a letter *Y*, you just copied a letter *Y*.” Now Hua knows this is how you write a letter *Y*. She knows this is a letter *Y*.

Mei Ran shows his letter to the teacher. The teacher says to Mei Ran: “This is a letter, you just copied a nice letter.” Only Mei Ran does not know this is how you write a letter *Y*. He does not know this is a letter *Y*.

**Control questions:**

At the end of the story, does Hua know this is how to write a letter *Y*?

At the end of the story, does Mei Ran know this is how to write a letter *Y*?

**Learning question:**

Who learned how to write a letter *Y* today, Hua or Mei Ran?

1. **Familiar content: Spelling**

Do you know how to spell the word *DOG*? Can you spell the word *DOG* for me?

These are Peng and Ying Ying. They cannot spell the word *DOG*. They just can’t.

Today in school, Peng and Ying Ying each put three letter blocks together: D-O-G. They spell just like the word *DOG*, don’t they?

Peng shows his blocks to the teacher. The teacher says to Peng: “This is a line of blocks, you just did a good job putting these blocks together.” Only Peng does not know this is how you spell the word *DOG*. He does not know this is the word *DOG*.

Ying Ying shows her Blocks to the teacher. The teacher says to Ying Ying: “This is the word *DOG*, you just spelled the word *DOG*.” Now Ying Ying knows this is how you spell the word *DOG*. She knows this is the word *DOG*.

**Control questions:**

At the end of the story, does Peng know this is how to spell the word *DOG*?

At the end of the story, does Ying Ying know this is how to spell the word *DOG*?

**Learning question:**

Who learned how to spell the word *DOG* today, Peng or Ying Ying?

1. **Unfamiliar content: Duel Identity**

Do you know how to write the number 4 in Japanese? This is how you write the number 4 in Japanese: し.

These are De Xi and Le Le. They cannot write 4 in Japanese. They just can’t.

Today in school, De Xi and Le Le each draw a hook: し. It looks just like the number 4 in Japanese, doesn’t it?

De Xi shows his hook to the teacher. The teacher says to De Xi: “This is the number 4 in Japanese, you just wrote a number 4 in Japanese.” Now De Xi knows this is how you write the number 4 in Japanese. He knows this is the number 4 in Japanese.

Le Le shows her hook to the teacher. The teacher says to Le Le: “This is a hook, you just drew a nice hook.” Only Le Le does not know this is how you write the number 4 in Japanese. She does not know this is a number 4 in Japanese.

**Control questions:**

At the end of the story, does De Xi know this is how to write the number 4 in Japanese?

At the end of the story, does Le Le know this is how to write the number 4 in Japanese?

**Learning question:**

Who learned how to write the number 4 in Japanese today, De Xi or Le Le?

1. **Unfamiliar content: Copy**

Do you know how to write the character for *hat* in ancient Chinese? This is how you write the character for *hat* in ancient Chinese: 冃.

These are Si Si and Yuan. They cannot write *hat* in ancient Chinese. They just can’t.

Today in school, Si Si and Yuan each copy a character from a book: 冃. It looks just like the character for *hat* in ancient Chinese, doesn’t it?

Si Si shows his character to the teacher. The teacher says to Si Si: “This is a character, you just copied a nice character.” Only Si Si does not know this is how you write *hat* in ancient Chinese. He does not know this is *hat* in ancient Chinese.

Yuan shows her character to the teacher. The teacher says to Yuan: “This is how you write *hat* in ancient Chinese, you just wrote *hat* in ancient Chinese.” Now Yuan knows this is how you write *hat* in ancient Chinese. She knows this is *hat* in ancient Chinese.

**Control questions:**

At the end of the story, does Si Si know this is how you write *hat* in ancient Chinese?

At the end of the story, does Yuan know this is how you write *hat* in ancient Chinese?

**Learning question:**

Who learned how to write *hat* in ancient Chinese today, Si Si or Yuan?

1. **Unfamiliar content: Spelling**

There is a planet called Emma in the Milky Way. People on Planet Emma speak Emma language. Do you know how to spell *pen* in Emma language? This is how you spell *Pen* in Emma language: *WUP*.

These are Bing and Sim Yee. They cannot spell *pen* in Emma language. They just can’t.

**Today in school, Bing and Sim Yee each put three letter blocks together: *WUP*.** They spell just like the word *pen* **in Emma language, don’t they?**

Bing shows his blocks to the teacher. The teacher says to Bing: “This is how you spell *pen* in Emma language, you just spelled *pen* in Emma language.” Now Bing knows this is how you spell *pen* in Emma language. He knows this is *pen* in Emma language.

Sim Yee shows her blocks to the teacher. The teacher says to Sim Yee: “This is a line of blocks, you just did a good job putting these blocks together.” Only Sim Yee does not know this is how you spell p*en* in Emma language. She does not know this is p*en* in Emma language.

**Control questions:**

At the end of the story, does Bing know this is how you spell Pen in Emma language?

At the end of the story, does Sim Yee know this is how you spell Pen in Emma language?

**Learning question:**

Who learned how to spell Pen in Emma language today, Bing or Sim Yee?

**Learning intention stories in Study 2**

1. **Discovery learning**

Do you know how to make green paint using other colors? Can you show it to me?

This is Ming. Ming cannot make green paint. He just can’t.

Today in school, Ming drops some blue paint in yellow paint by accident while painting. Oops! Look what happened. The two colors make green. “So that’s how you make green paint,” Ming says. When the teacher asks Ming: “Can you make green paint?” Ming says: “Yes, I can. I can mix yellow paint and blue paint together and make green.” Ming can make green paint now.

**Control questions:**

At the beginning of the story, can Ming make green paint?

At the end of the story, can Ming make green paint?

**Intention question:**

Did Ming try to learn how to make green paint today?

**Learning question:**

Did Ming learn how to make green paint today?

1. **Failed learning**

Do you know how to play rock-paper-scissors? Shall we play?

This is Ning. Ning cannot play rock-paper-scissors. She just can't.

The teacher can play rock-paper-scissors. Today in school the teacher and other kids in the class are playing rock-paper-scissors. Ning does not watch or listen to them playing or tries to find out how. She just sits there and plays by herself. The teacher says: “Rock beats scissors, paper beats rock, and scissors beat paper.” Then the teacher asks Ning: “Can you play rock-paper-scissors?” Ning says: “No, I can't.” Ning cannot play rock-paper-scissors.

**Control questions:**

At the beginning of the story, can Ning play rock-paper-scissors?

At the end of the story, can Ning play rock-paper-scissors?

**Intention question:**

Did Ning try to learn how to play rock-paper-scissors?

**Learning question:**

Did Ning learn how to play rock-paper-scissors today?

1. **Implicit learning**

Do you know how to sing the *Birthday Song*? Can you sing the *Birthday Song*?

This is Rui. Rui cannot sing the *Birthday Song*. He just can't.

Today in school, some kids are singing the *Birthday Song*. Rui covers his ears and tries very hard not to listen to that song. The kids sing like this: “♫ *Happy birthday to you, happy birthday to you.*” Later when Rui begins to sing, he sings the *Birthday Song*. He sings like this: “♫ *Happy birthday to you, happy birthday to you.*” Rui can sing the *Birthday Song*.

**Control questions:**

At the beginning of the story, can Rui sing the *Birthday Song*?

At the end of the story, can Rui sing the *Birthday Song*?

**Intention question:**

Did Rui try to learn the birthday song, or did he try not to learn the *Birthday Song*?

**Learning question:**

Did Rui learn how to sing the *Birthday Song* today?

1. **Resistance to learning**

Do you know how to make a paper plane? Can you make a paper plane for me?

This is Sue. Sue cannot make a paper plane. She just can't.

Her teacher can make a paper plane. Today in school, her teacher is making a paper plane. Sue covers her eyes and tries very hard not to look. Then her teacher asks Sue: “Can you make a paper plane?” Sue says: “No, I can’t.” Sue cannot make a paper plane.

**Control questions:**

At the beginning of the story, can Sue make a paper plane?

At the end of the story, can Sue make a paper plane?

**Intention question:**

Did Sue try to learn how to make a paper plane, or did she try not to learn how to make a paper plane?

**Learning question:**

Did Sue learn how to make a paper plane today?
